# Supplementary material for: The Chicken cGAS–STING Pathway Exerts Interferon-Independent Antiviral Function via Cell Apoptosis
Source: Animals (Basel). 2023 Aug 9;13(16):2573. doi: 10.3390/ani13162573 (PMC10451998; doi:10.3390/ani13162573)
Supplement: Supplementary file 1 [file animals-13-02573-s001.zip › Supplementary Tables.pdf]

Supplementary Table S1. PCR primers used for the construction of chSTING mutants

Primer Names	Sequences (5′-3′)
chSTING pLxVS sub S366A-F	CCGAGCTACTGATCGCCTCCTCCGACCTGC
chSTING pLxVS sub S365A-R	GCAGGTCGGAGGAGGCGATCAGTAGCTCGG
chSTING-△CTT-F	CAGCAGCAGCGCGAGGATATCACCAGCTAC
chSTING-△CTT-R	GTAGCTGGTGATATCCTCGCGCTGCTGCTG
chSTING S366A-F	CAGGTCGGAGGAGGCGACCTGCAGGCTG
chSTING S365A-R	CAGCCTGCAGGTCGCCTCCTCCGACCTG

Supplementary Table S2. Primers for qPCR in this study

Primer Names	Sequences (5′-3′)
hRPL32-F	CAACATTGGTTATGGAAGCAACA
hRPL32-R	TGACGTTGTGGACCAGGAACT
hISG56-F	CGCTATAGAATGGAGTGTCCA
hISG56-R	TTTCCTCCACACTTCAGCA
hISG60-F	AGTCTAGTCACTTGGGGAAAC
hISG60-R	ATAAATCTGAGCATCTGAGAGTC
hIL-8-F	GTTTTTGAAGAGGGCTGAGAATTC
hIL-8-R	CATGAAGTGTTGAAGTAGATTTGCTTG
hIFN-β-F	TGGGAGGATTCTGCATTACC
hIFN-β-R	CAGCATCTGCTGGTTGAAGA
hTNF-α-F	TGGCCCAGGCAGTCAGA
hTNF-α-R	GGTTTGCTACAACATGGGCTACA
chGAPDH-F	AGGGTGGTGCTAAGCGTGTTAT
chGAPDH-R	CAGCAGCCTTCACTACCCTCTT
chOASL-F	CTGTCCTTCGGAGTCAGCATCA
chOASL-R	TCAGCAGCTCCAGTGCATACTT
chIFN-β-F	ATCTTCGTCACCAGGATGCCAA
chIFN-β-R	CGTGCCTTGGTTTACGAAGCAT
chIL-8-F	GGACGCTGGTAAAGATGGGGAA
chIL-8-R	CAGAATTGAGCTGAGCCTTGGC
SMV/VACV-detection-F	TCTGATGTTGTTGTTCGCTGCT
SMV/VACV-detection-R	TCCATCTCCCTCTGGACCGCAT
