# Supplementary material for: The Chicken cGAS–STING Pathway Exerts Interferon-Independent Antiviral Function via Cell Apoptosis
Source: Animals (Basel). 2023 Aug 9;13(16):2573. doi: 10.3390/ani13162573 (PMC10451998; doi:10.3390/ani13162573)
Supplement: Supplementary file 1 [file animals-13-02573-s001.zip › Western blotting raw data.pdf]

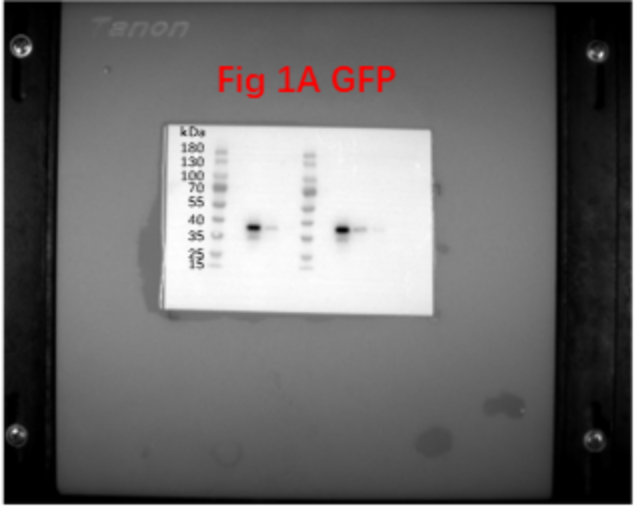
Fig 1A GFP
kDa
180
130
100
70
55
40
35
25
15

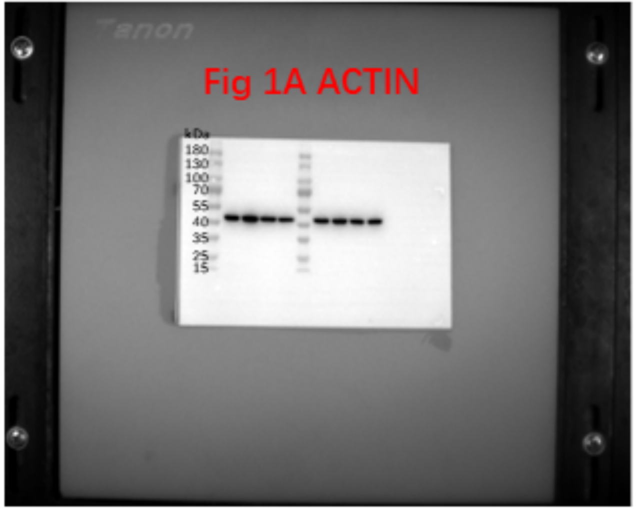
Fig 1A ACTIN
kDa
180
130
100
70
55
40
35
25
15

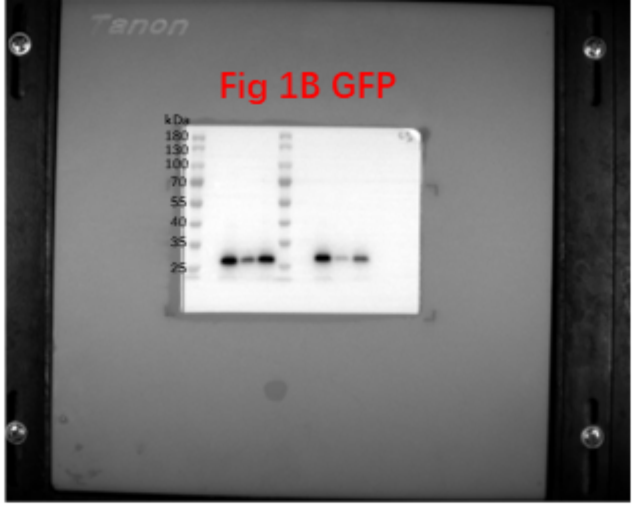
Fig 1B GFP
kDa
180
130
100
70
55
40
35
25

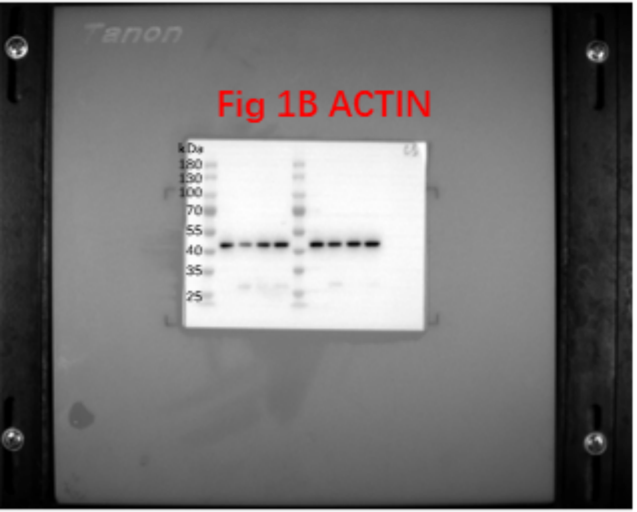
Fig 1B ACTIN
kDa
180
130
100
70
55
40
35
25

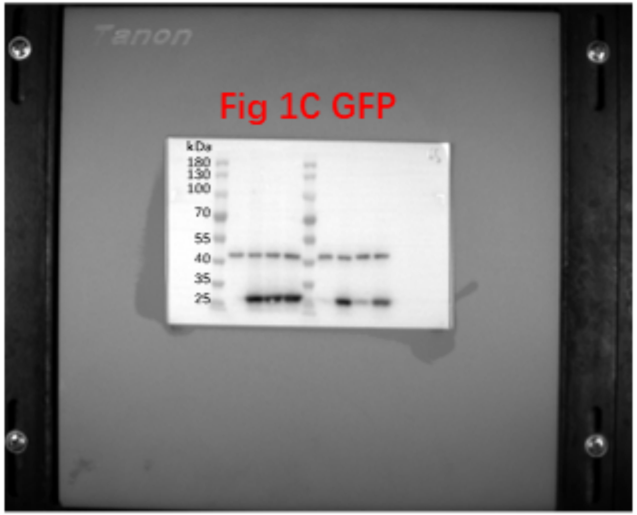
Fig 1C GFP
kDa
180
130
100
70
55
40
35
25

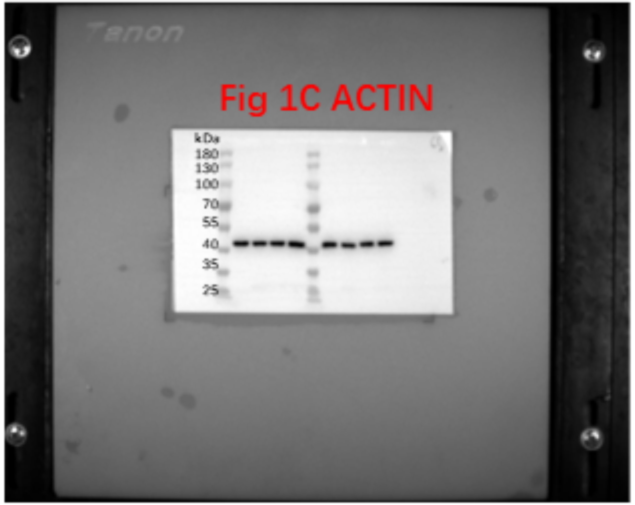
Fig 1C ACTIN
kDa
180
130
100
70
55
40
35
25

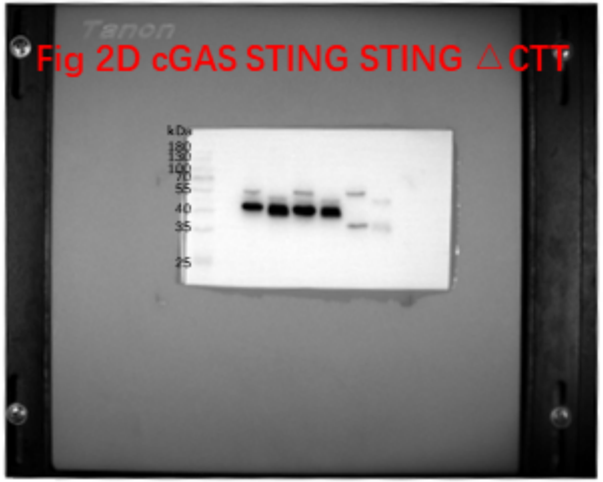
Tanon
Fig 2D cGAS STING STING △CTT
kDa
180
130
100
70
55
40
35
25

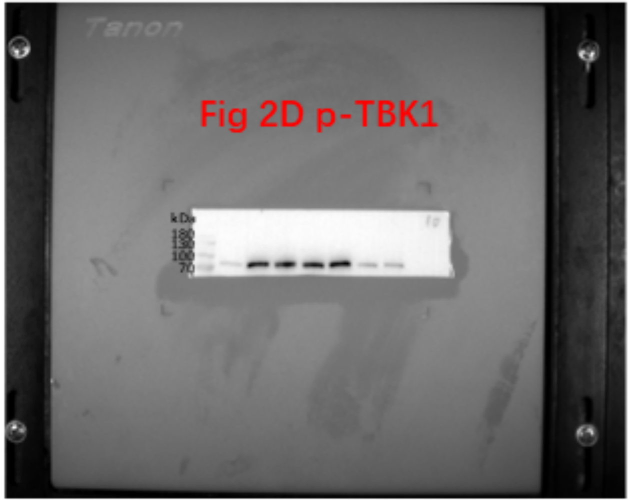
Tanon
Fig 2D p-TBK1
kDa
180
130
100
70

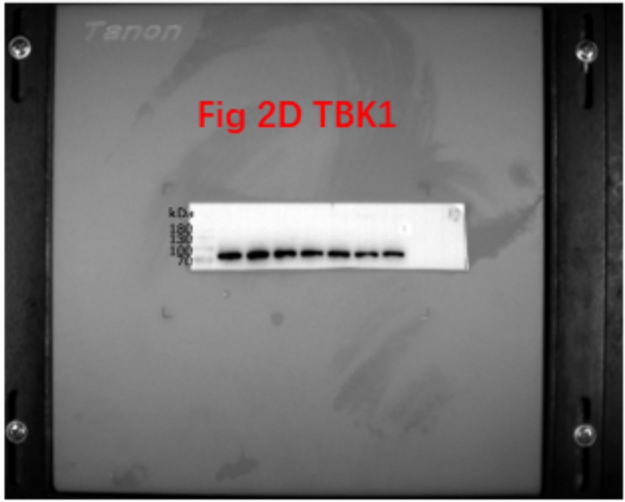
Tanon
Fig 2D TBK1
kDa
180
130
100
70

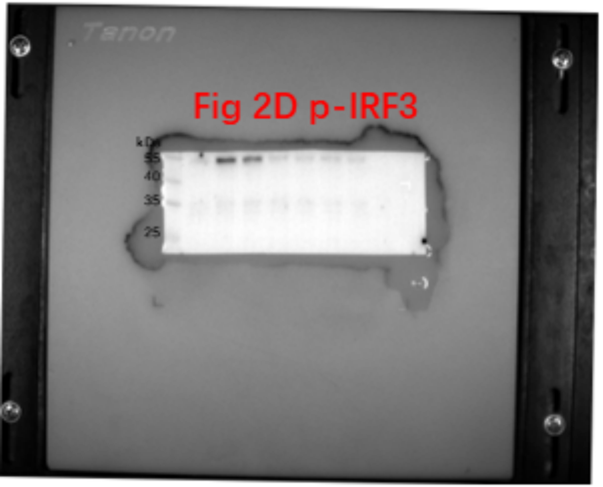
Tanon
Fig 2D p-IRF3
kDa
55
40
35
25

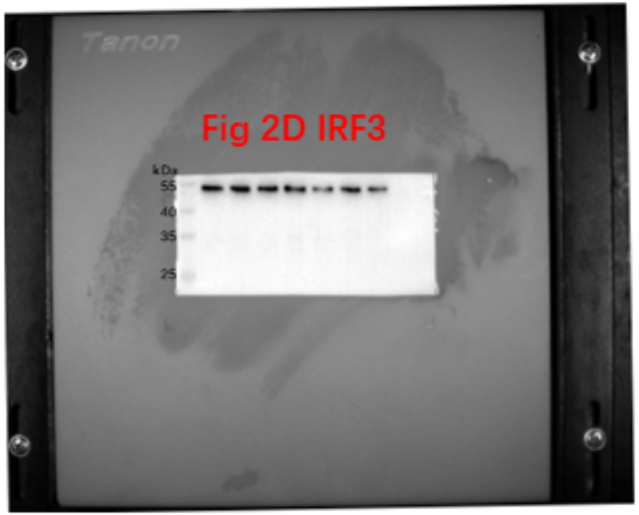
Tanon
Fig 2D IRF3
kDa
55
40
35
25

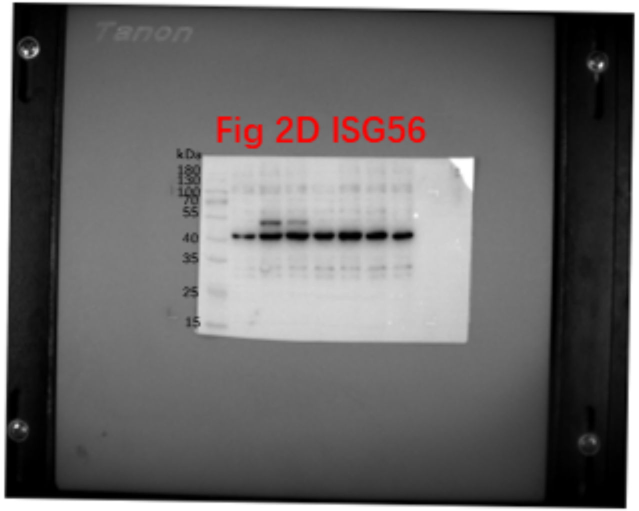
Tanon
Fig 2D ISG56
kDa
180
130
100
70
55
40
35
25
15

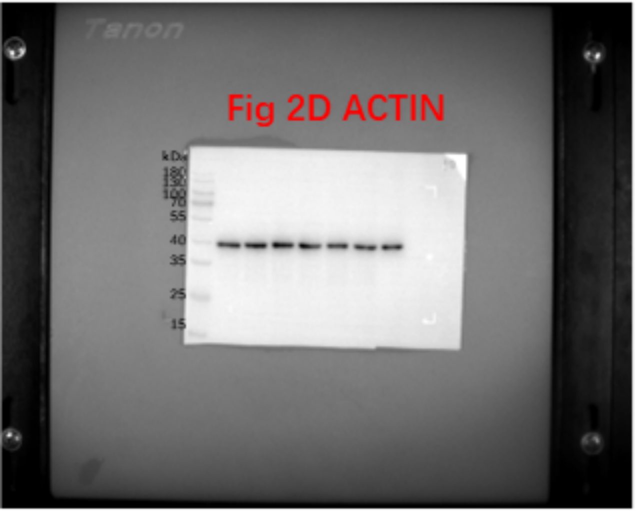
Fig 2D ACTIN

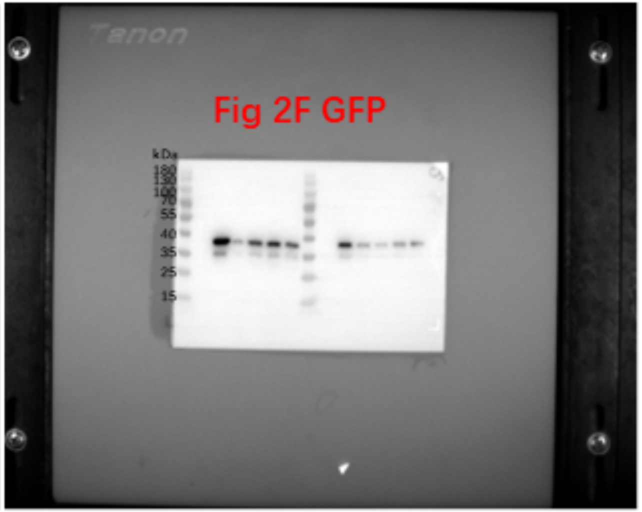
Fig 2F GFP

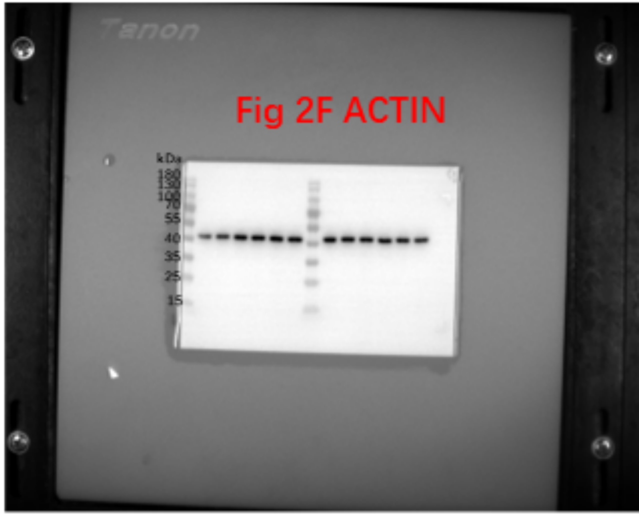
Fig 2F ACTIN

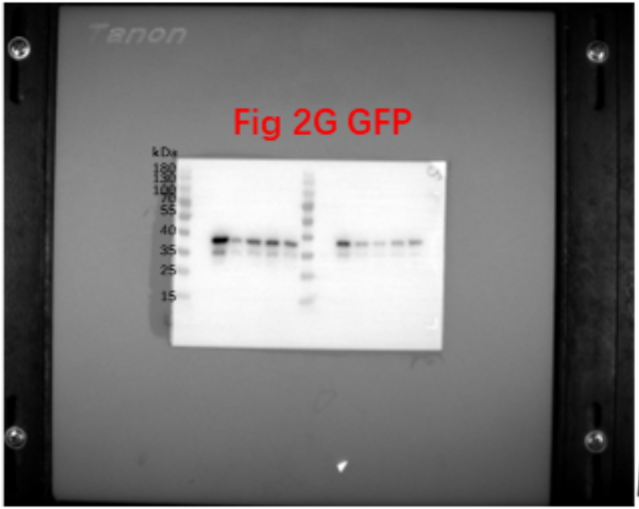
Fig 2G GFP

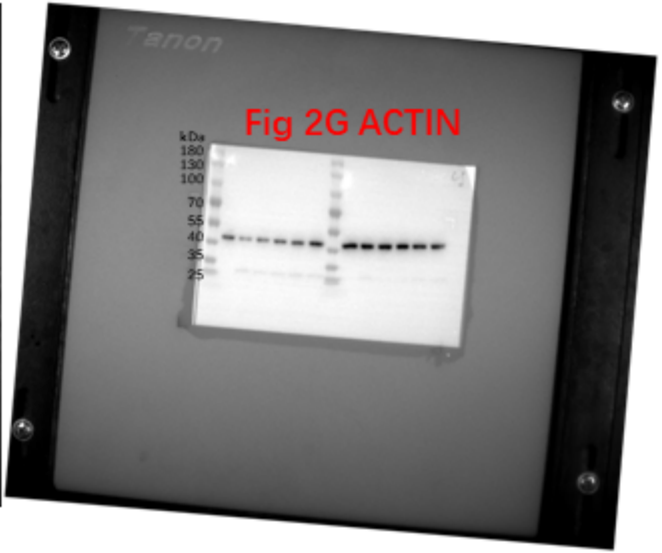
Fig 2G ACTIN

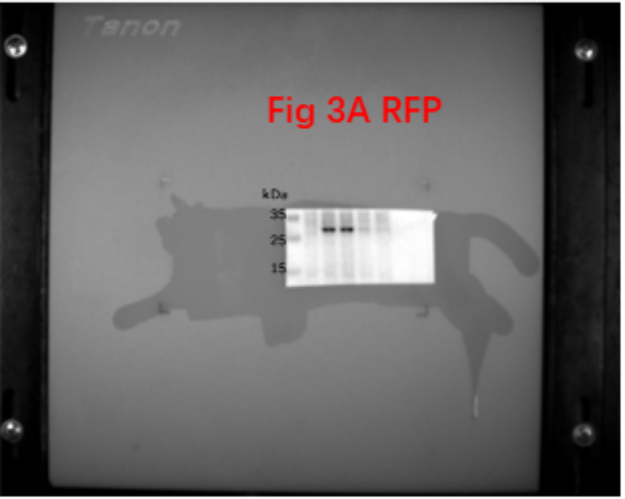
Tanon
Fig 3A RFP
kDa
35
25
15

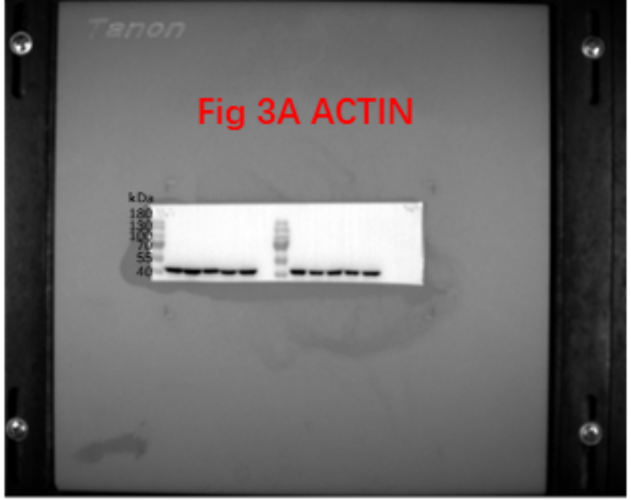
Tanon
Fig 3A ACTIN
kDa
180
130
100
70
55
40

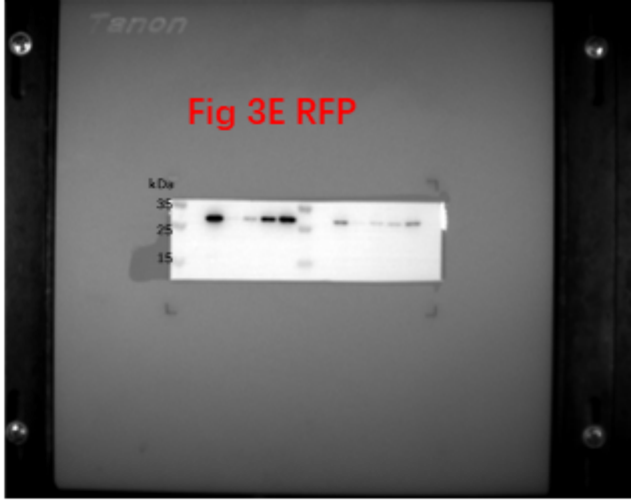
Tanon
Fig 3E RFP
kDa
35
25
15

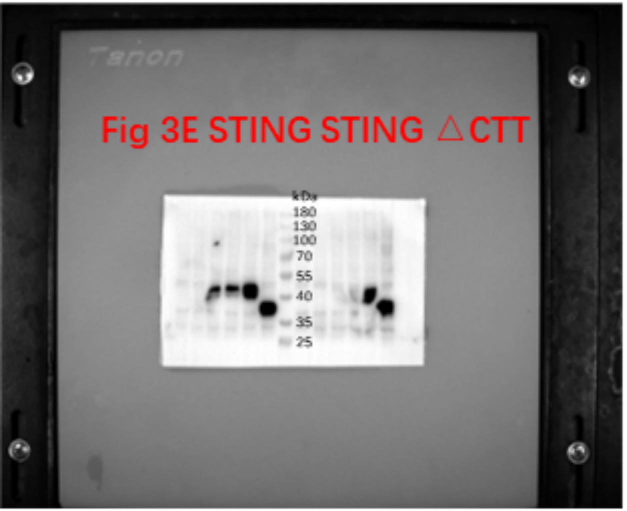
Tanon
Fig 3E STING STING △CTT
kDa
180
130
100
70
55
40
35
25

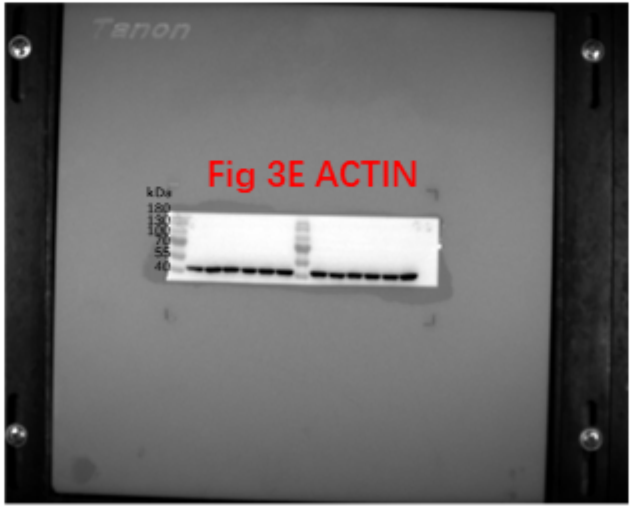
Tanon
Fig 3E ACTIN
kDa
180
130
100
70
55
40

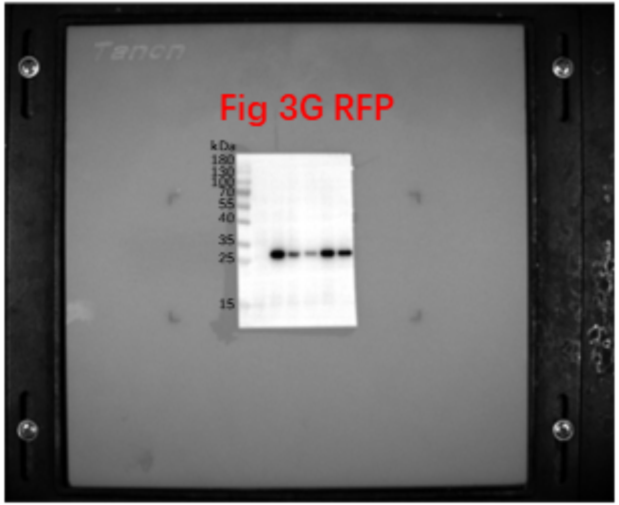
Fig 3G RFP
kDa
180
130
100
70
55
40
35
25
15

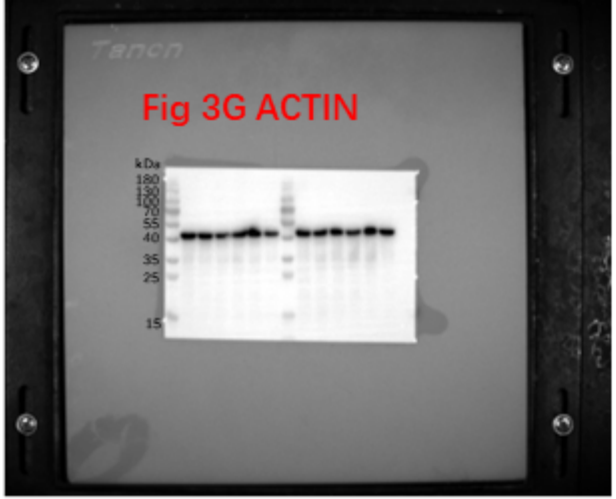
Fig 3G ACTIN
kDa
180
130
100
70
55
40
35
25
15

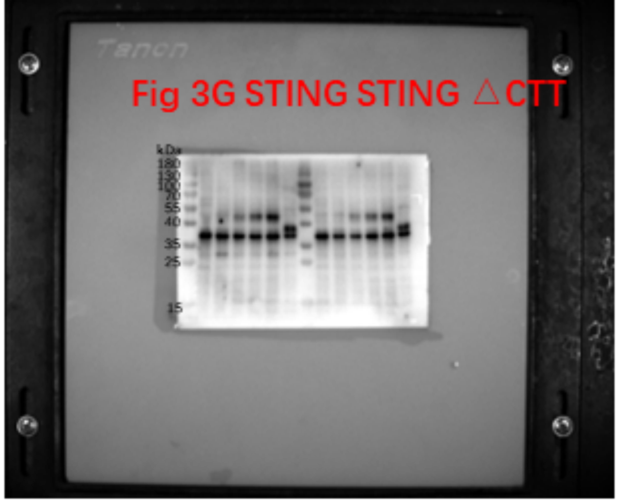
Fig 3G STING STING △CTT
kDa
180
130
100
70
55
40
35
25
15

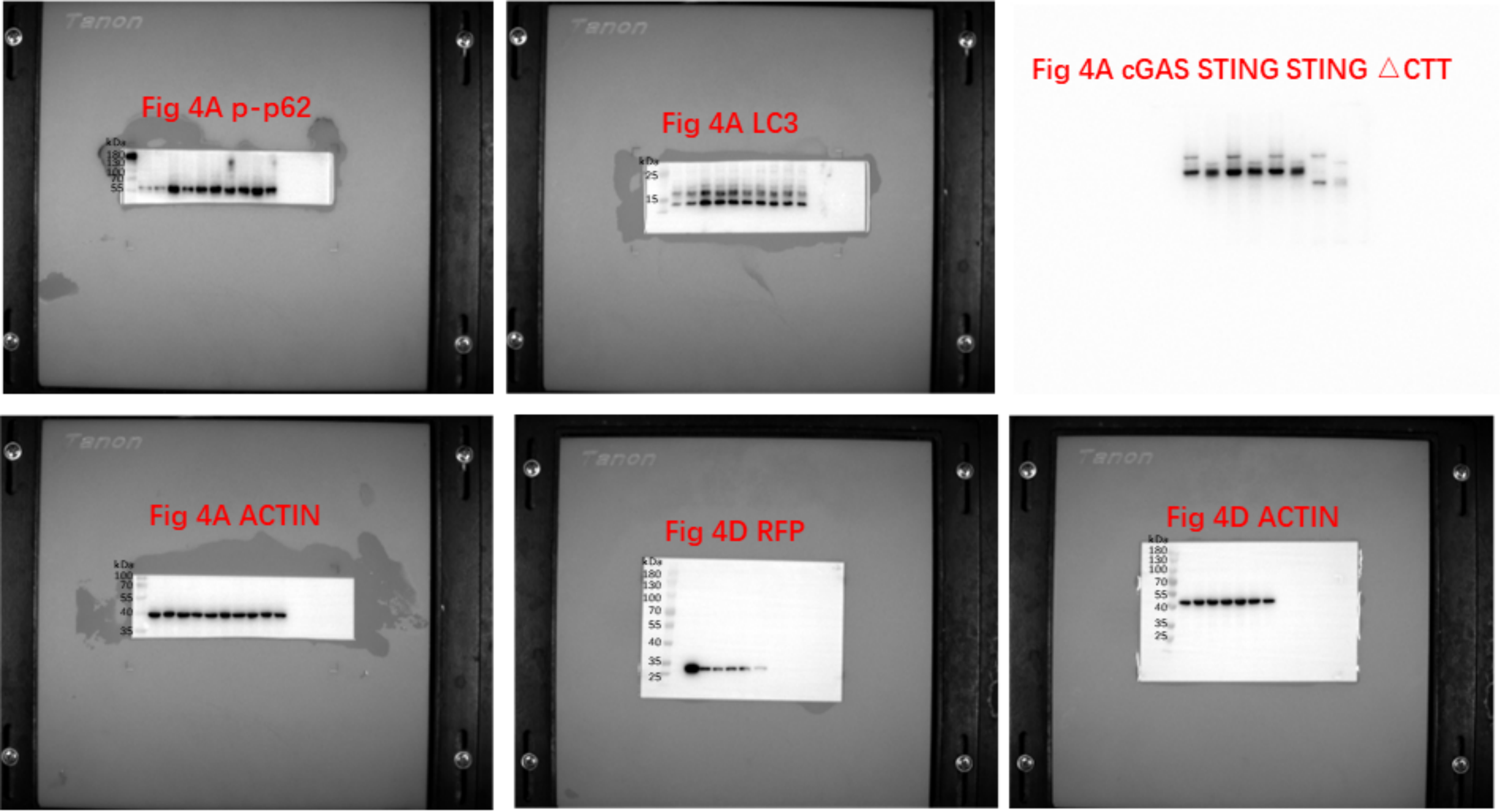
Fig 4A p-p62
Fig 4A LC3
Fig 4A cGAS STING STING △CTT
Fig 4A ACTIN
Fig 4D RFP
Fig 4D ACTIN

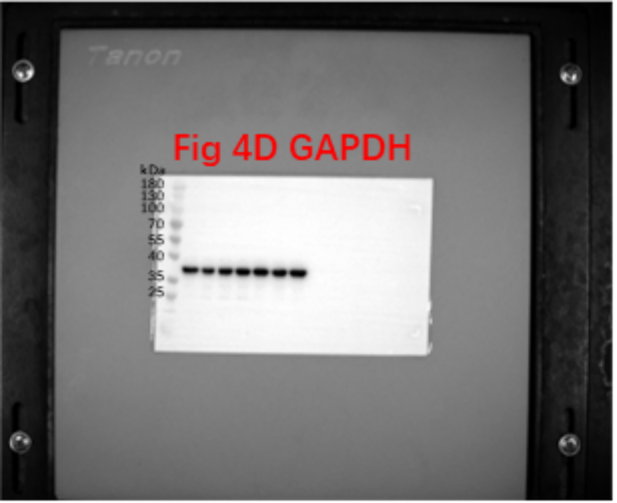
Tanon
Fig 4D GAPDH
kDa
180
130
100
70
55
40
35
25

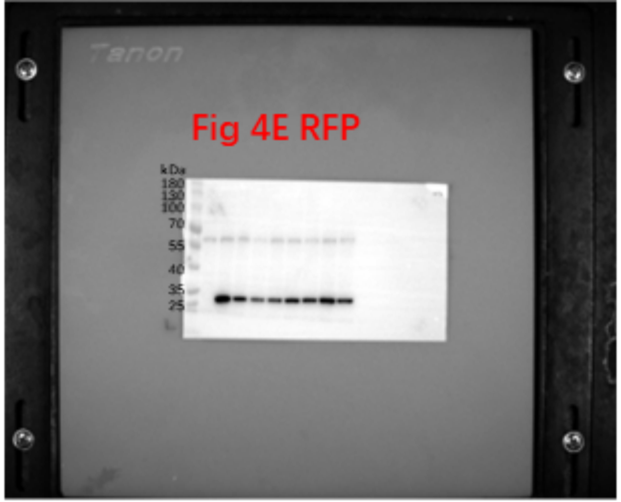
Tanon
Fig 4E RFP
kDa
180
130
100
70
55
40
35
25

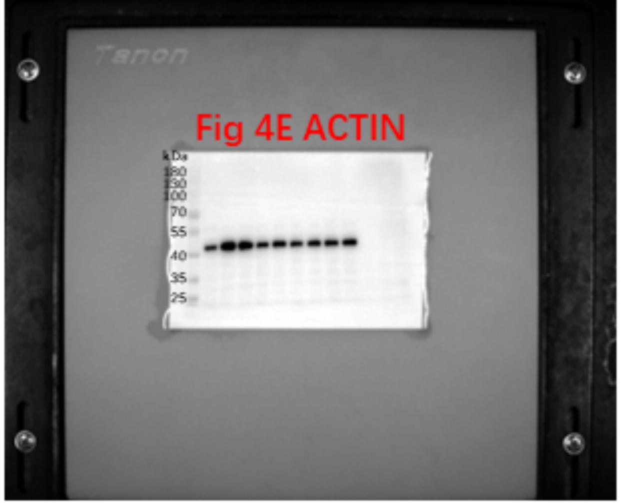
Tanon
Fig 4E ACTIN
kDa
180
130
100
70
55
40
35
25

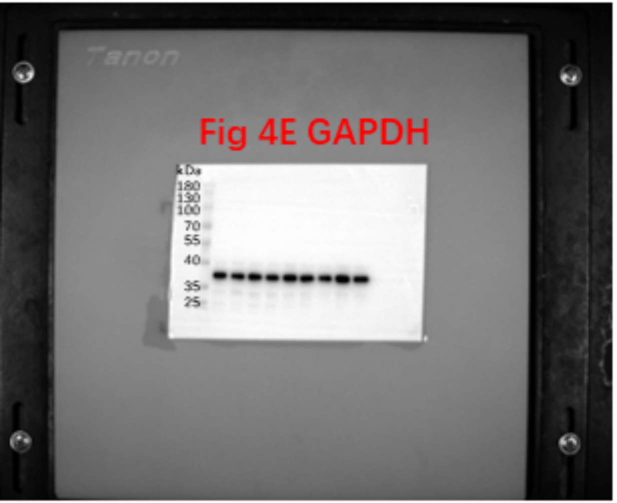
Tanon
Fig 4E GAPDH
kDa
180
130
100
70
55
40
35
25

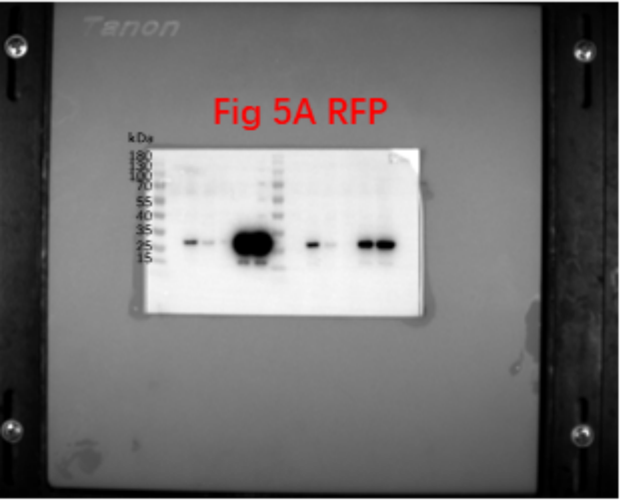
Fig 5A RFP

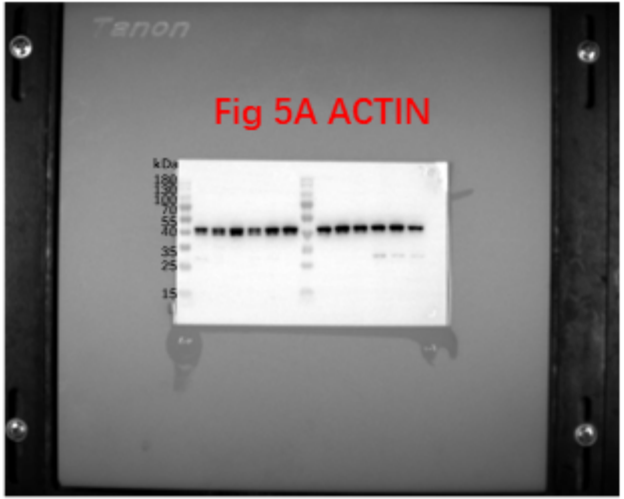
Fig 5A ACTIN

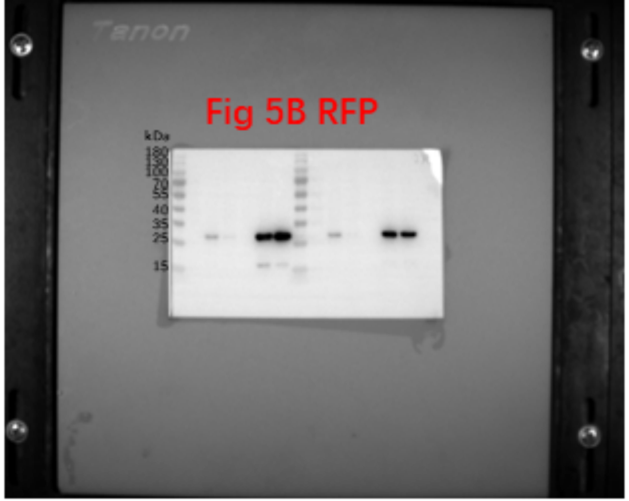
Fig 5B RFP

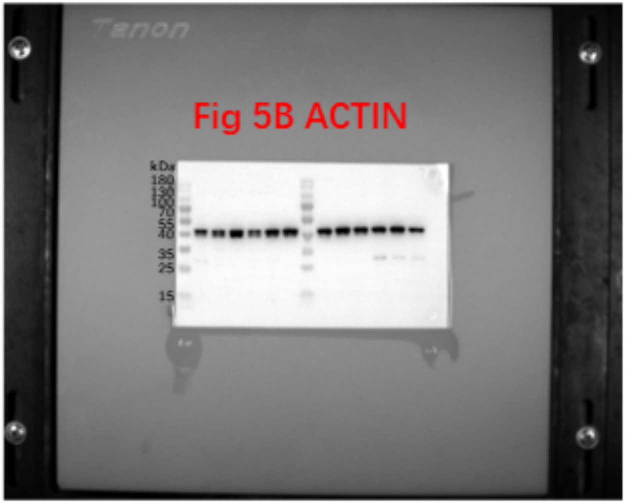
Fig 5B ACTIN

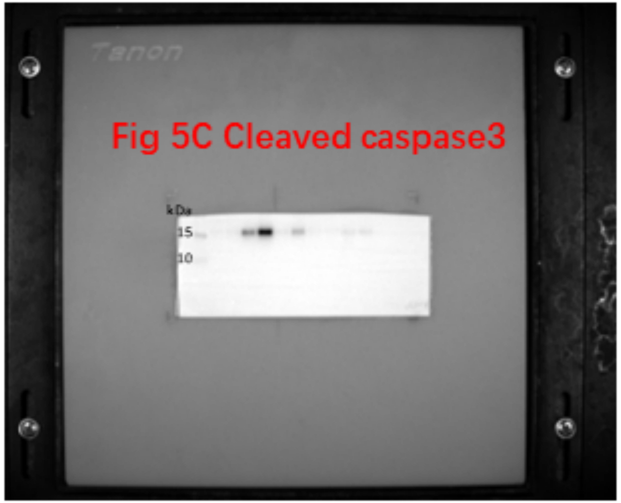
Fig 5C Cleaved caspase3

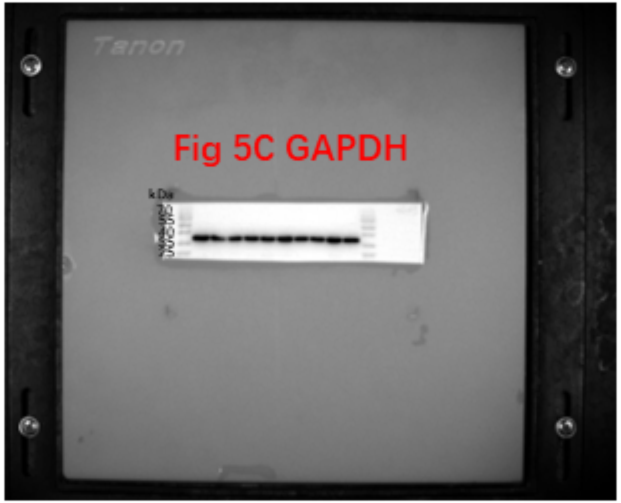
Fig 5C GAPDH

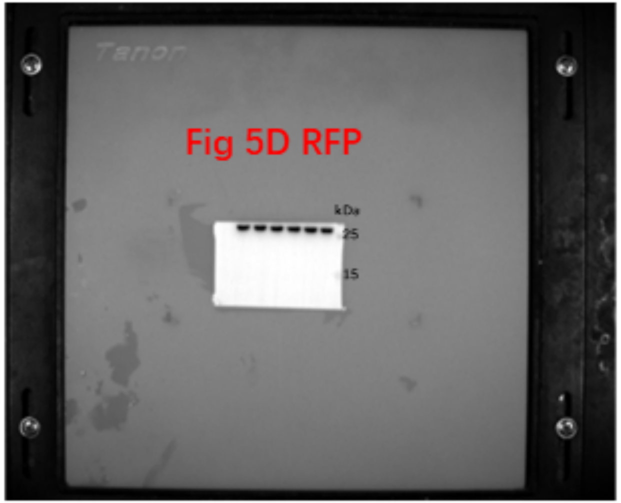
Tanon
Fig 5D RFP
kDa
25
15

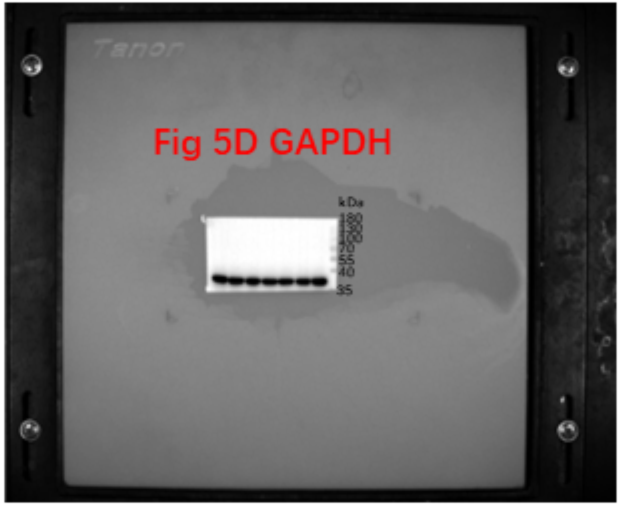
Tanon
Fig 5D GAPDH
kDa
180
130
100
70
55
40
35
