# Supplementary figures and images for: The Chicken cGAS–STING Pathway Exerts Interferon-Independent Antiviral Function via Cell Apoptosis
Source: Animals (Basel). 2023 Aug 9;13(16):2573. doi: 10.3390/ani13162573 (PMC10451998; doi:10.3390/ani13162573)

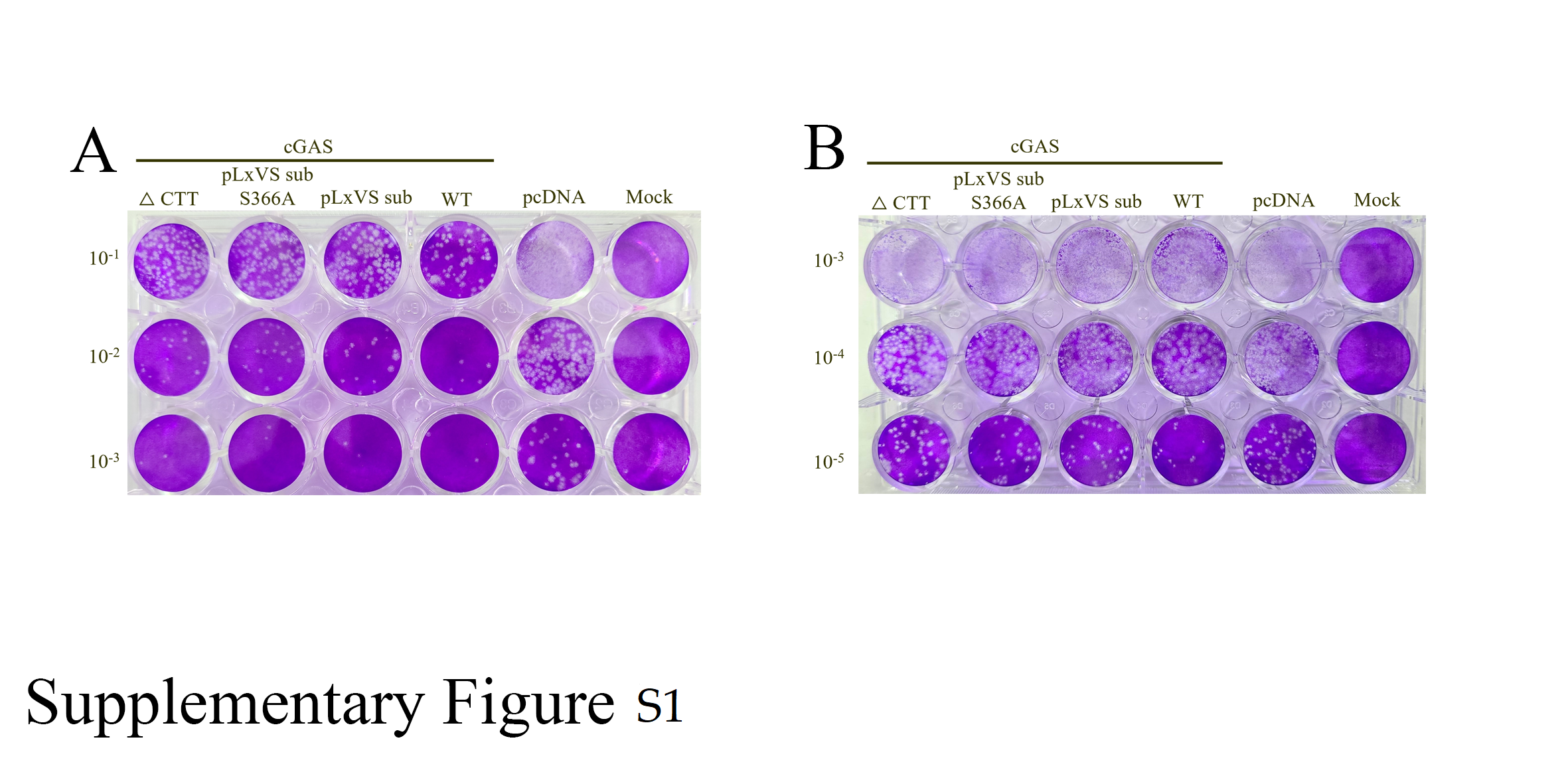

Supplement: Supplementary file 1 [file animals-13-02573-s001.zip › Supplementary Figure S1.tif]
